# Racial/ethnic inequities in the associations of allostatic load with all-cause and cardiovascular-specific mortality risk in U.S. adults

**DOI:** 10.1371/journal.pone.0228336

**Published:** 2020-02-13

**Authors:** Luisa N. Borrell, Elena Rodríguez-Álvarez, Florence J. Dallo

**Affiliations:** 1 Department of Epidemiology & Biostatistics, Graduate School of Public Health & Health Policy, City University of New York, New York, New York, United States of America; 2 OPIK-Research Group for Social Determinants of Health and Demographic Change, University of the Basque Country (UPV/EHU), Bizkaia, Spain; 3 Department of Surgery, Medical and Social Science, University of Alcalá, Madrid, Spain; 4 Department of Nursing I, Faculty of Medicine and Nursing, University of the Basque Country (UPV/EHU), Bizkaia, Spain; 5 Department of Public and Environmental Wellness, School of Health Sciences, Oakland University, Rochester, Michigan, United States of America; University of Alberta, CANADA

## Abstract

Non-Hispanic blacks have higher mortality rates than non-Hispanic whites whereas Hispanics have similar or lower mortality rates than non-Hispanic blacks and whites despite Hispanics’ lower education and access to health insurance coverage. This study examines whether allostatic load, a proxy for cumulative biological risk, is associated with all-cause and cardiovascular (CVD)-specific mortality risks in US adults; and whether these associations vary with race/ethnicity and further with age, sex and education across racial/ethnic groups. Data from the third National Health and Nutritional Examination Survey (NHANES III, 1988–1994) and the 2015 Linked Mortality File were used for adults 25 years or older (n = 13,673 with 6,026 deaths). Cox proportional hazards regression was used to estimate the associations of allostatic load scores (2 and ≥3 relative to ≤1) with a) all-cause and b) CVD-specific mortality risk among NHANES III participants before and after controlling for selected characteristics. Allostatic load scores are associated with higher all-cause and CVD-specific mortality rates among U.S. adults aged 25 years or older, with stronger rates observed for CVD-specific mortality. All-cause mortality rates for each racial/ethnic group differed with age and education whereas for CVD-specific mortality rates, this difference was observed for sex. Our findings of high allostatic load scores associated with all-cause and CVD-specific mortality among US adults call attention to monitor conditions associated with the allostatic load’s biomarkers to identify high-risk groups to help monitor social inequities in mortality risk, especially premature mortality.

## Introduction

In the United States (U.S.), there has been an increase in all-cause mortality rates between 2016 and 2017 (age-adjusted rates: 728.8/100,000 and 731.9/100,000, respectively).[1, 2] These estimates vary by race/ethnicity: When compared to non-Hispanic whites, non-Hispanic blacks exhibited higher mortality rates whereas Hispanics had lower rates after adjusting for age.[1, 3] However, these rates were lower for women than men regardless of race/ethnicity.[1] The lower rates observed among Hispanics has been attributed to the Hispanic paradox or an advantage on morbidity and mortality outcomes.[4] This advantage is observed despite of non-Hispanic blacks and Hispanics sharing a minority status in the U.S. and Hispanics being less educated and less likely to have insurance coverage than non-Hispanic blacks and whites.[5, 6] This paradox suggests that socioeconomic position may not fully explain racial/ethnic mortality inequities. Therefore, there may be other mechanisms for the non-Hispanic black-white gap. While age-adjusted mortality rates for cause-specific mortality in the U.S. are not available according to race/ethnicity,[1, 7] heart disease mortality has decreased since 2000 (256.6/100,000 in 2000; 179.1/100,000 in 2010; and 165.0/100,000 in 2017).[2]

A potential explanation for this gap is the embodiment of cumulative experiences of social inequality and racism among non-Hispanic blacks leading to chronic stress or the weathering hypothesis.[8] Evidence suggests that these experiences may disrupt allostasis or the ability (or lack thereof) of physiological systems to respond and/or adapt to prolonged or repeated stress.[9, 10] While there has not been a consistent definition to capture this physiological change, allostatic load, a summary index of multi-system risk,[11] has been associated with an increased mortality risk in the U.S.[12–16] and elsewhere.[17–19] However, only one study has examined allostatic load and cause-specific mortality (i.e., circulatory, neoplasm, respiratory, mental & behavioral, digestive and other) and found no association.[17] In the U.S., Geronimus et al. found that non-Hispanic blacks exhibited a higher allostatic load mean score than non-Hispanic whites.[20] Interestingly, this study suggested that allostatic load mean score for non-Hispanic blacks was comparable to non-Hispanic whites, who were 10 years older.[20] This finding is consistent with the “weathering hypothesis”[8] or a premature health deterioration that may lead to early onset of chronic diseases and/or premature death (i.e., death before 65 years of age). In the U.S., mortality rates increased for younger adults (<64 years) between 2011 and 2017 regardless of race and ethnicity.[3]

Previous studies suggest that high allostatic load may be associated with low socioeconomic position in the U.S.[16, 21] and elsewhere.[22–24] For instance, Seeman et al. showed an inverse association between allostatic load scores and educational attainment among U.S. adults aged 70 to 79 years.[16] Using NHANES data, Seeman et al. also found that the probability of having a low risk score for allostatic load increases with educational attainment among U.S. adults aged 20 years or older.[21] However, this pattern differs by race/ethnicity with non-Hispanic blacks and Mexican Americans showing a lower probability of having a low score as education increases compared with non-Hispanic whites. Therefore, it is imperative to examine the intertwined relationship among age, education and race/ethnicity as they relate to allostatic load and mortality risk in the U.S.

Using data from the National Health and Nutrition Examination Survey (NHANES) III linked to the 2015 mortality file, we examine whether allostatic load, using a cumulative biological risk index,[12, 21] is associated with all-cause and cardiovascular (CVD)-specific mortality risks in U.S. adults 25 years of age or older; and whether these associations vary with race/ethnicity and further with age, sex and education across racial/ethnic groups.

## Methods

We used publicly available data from NHANES III and the 2015 Linked Mortality File (LMF) with follow up through December 31^st^, 2015 from the Centers for Disease Control and Prevention, National Center for Health Statistics (NCHS) website.[25] NHANES III is a national survey conducted to assess the health status of the civilian non-institutionalized US population using a stratified multistage probability sample. More details on the sample design in NHANES III have been reported elsewhere.[26] Briefly, NHANES used a stratified multistage probability design of the total civilian noninstitutionalized population in the 50 states of the US. The sampling design consisted of three stages: 1) primary sampling units, 2) segments composed of city or suburban blocks, or combinations of blocks, and 3) households and certain types of groups quarters, such as dormitories. All households and eligible group quarters in the sample segments were listed, and a subsample was designated for screening to identify potential sample persons. NHANES III oversampled younger and older ages groups, as well as non-Hispanic blacks and Hispanics to ensure precise estimates. For this analysis, NHANES III publicly available household adult, examination, and laboratory datasets were linked with the 2015 public-use LMF.[27] These datasets are matched by NCHS using a standard methodology described elsewhere.[13] Briefly, the NCHS probabilistic matching method used an algorithm based on information for social security number, first name, middle initial, last name or surname, month, day and year of birth, sex, father’s surname, state of birth, race, state of residence, and marital status.

We specified mortality status using the underlying cause of death (UCOD) based on the International Classification of Diseases (ICD), Ninth and Tenth Revisions,[28] provided in the LMF dataset. For analytical purposes, we used the mortality status provided in the 2015 LMF dataset to determine all-cause mortality and the UCOD values associated with ICD10 codes I00-I09, I11-I13, I20-I51, and I60-I69 to specify CVD-specific deaths. Time to death was specified in person-years from the date of the NHANES III interview through December 31, 2015: For those who died, we used the interview date through the date of death; and for those alive or assumed to be alive, from interview date to the end of follow-up.[29]

Consistent with previous studies,[12, 21] the allostatic load score or cumulative biological risk profile was developed using biomarkers associated with specific organs and tissues: Cardiovascular disease and atherosclerosis (systolic blood pressure, diastolic blood pressure, pulse, high density lipoprotein and total cholesterol); metabolic syndrome (waist-to-hip ratio and glycated hemoglobin); and inflammation (C-reactive protein and albumin).[9, 15, 30] We used at-risk cut points for each biomarker collected as part of the lab data from the NHANES III (1988–1994): albumin < 3.8 mg/dl; C-reactive protein ≥ 0.3 mg/dl; total cholesterol ≥ 240 mg/dl; High Density Lipoprotein (HDL) < 40 mg/dl; glycated hemoglobin ≥ 6.4%; male waist-to-hip ratio > 0.90/female waist-to-hip ratio > 0.85; systolic blood pressure ≥ 140 mmHg; diastolic blood pressure ≥ 90 mmHg; and resting heart rate ≥ 90 beats/minute.[12, 21] Each biomarker was assigned a value of 1 if its’ value was at or above the at-risk cut point and a value of 0 otherwise. We then summed the biomarker values to obtain the allostatic load score for each individual. The allostatic load score has a range of 0 to 9 and using its distribution in the total population, it was categorized as ≤1, 2, and ≥3. We treated each biomarker with an equal weight although each biomarker may not contribute equally to the allostatic load score. However, while this specification of allostatic load score provides an adequate estimate for the true effect of allostatic load on the outcome, the estimate may underestimate the true effect.[31]

We included socio-demographic and health behavior characteristics considered by previous studies [12, 14, 16–18] as covariates. Age was specified as a continuous and a categorical (25–44, 45–64, or ≥65 years of age) variable. Sex was considered as collected by NHANES (male/female). Race/ethnicity was self-reported by survey participants. For analytical purposes, we included non-Hispanic white, non-Hispanic black, and Mexican American (hereafter, non-Hispanic black and non-Hispanic white will be referred to as black and white, respectively). Marital status was specified as married, divorced, single and widowed. Education attainment was collected as the highest year of education from 0 to 17 years and was categorized as less than a high school diploma/general equivalency diploma (GED), high school diploma/GED, and more than a high school diploma/GED. Total family 12-month income during the past year was collected as a continuous variable with increments of $999.99 up to $49,999 and $50,000 or over. Income was further categorized as <$14,999, $15,000–$24,999, ≥$25,000 and missing.

Of the 20,050 records for US adults aged 17 or older in the household adult NHANES III interview, we excluded records for individuals who were ineligible for follow-up (n = 452); were younger than 25 years of age (n = 2,733); reported a race/ethnicity as “other” (n = 625); did not have information on education (n = 177); did not have information on follow up time (n = 9); had a weight less than zero (n = 1,537); did not have information on mortality status (n = 48); and had an allostatic load score of less than five (n = 796). These exclusions yielded an analytical sample of 13,673, with 6,026 deaths (including 1,906 for CVD-specific causes) and approximately 283,151,900.1 person-years (mean = 20.37, SE = 0.22; median = 22.75, range: 0.08 to 27.17 years).

## Statistical analysis

We calculated descriptive statistics for selected characteristics of the population according to allostatic load score and for the total population. Specifically, we presented 1) the distribution of the selected covariates according to allostatic load categories and for the total population; 2) the prevalence of allostatic load score categories; and 3) the means and standard errors for each biomarker and the allostatic load score according to death status and for the total population. We also calculated death rates for all-cause and CVD-specific mortality per 100,000 person-years according to allostatic load score categories.

We used Cox proportional hazards regression to estimate hazard ratios (HRs) and 95% confidence intervals (CIs) and quantify the associations of allostatic load scores (2 and ≥3 relative to a score of ≤1) with a) all-cause and b) CVD-specific mortality risk among NHANES III participants before and after controlling for selected characteristics. Specifically, we adjusted for age, sex, race/ethnicity, education and income. Marital status was not considered during the adjustment as it did not seem to affect these associations. We examined the proportional hazards assumption using the Schoenfeld residuals [32] and non-violation was observed that the hazards for any two individuals was constant over time. We compared the allostatic load score categories for all-cause and CVD-specific mortality risks using Kaplan-Meier survival curves and log-rank tests. In models for CVD-specific mortality, deaths attributed to other causes were treated as censored at the time the CVD-specific death occurred. To determine heterogeneity of these associations with race/ethnicity and further with age, sex, and education across racial/ethnic groups, interaction terms between 1) race/ethnicity and allostatic load score; and 2) race/ethnicity, allostatic load score and each covariate, were tested in the fully adjusted model. To avoid multicollinearity, each interaction was tested in a separate model.

All data management procedures were conducted with SAS software (SAS Institute, Cary, NC). Statistical analyses were conducted with SUDAAN software (RTI International, Research Triangle Park, NC). SUDAAN accounts for the complex sampling design and yields unbiased standard error estimates. Sample sizes presented in Table 1 were un-weighted, but all other estimates (proportions, standard errors, and HRs with their 95% CIs) were weighted.

**Table 1:** Distribution^a^ of selected socio-demographic and health-related characteristics according to allostatic load categories for adults 25 years of age or older: NHANES III-Linked Mortality File, 2015.

	Allostatic Load Score Categories			
	≤1 (n = 4,699)	2 (n = 3,475)	≥3 (n = 5,499)	Total (n = 13,673)
**Socio-Demographic**^**b**^				
Age				
25–44	70.2 (1.75)	47.3 (1.28)	28.9 (1.46)	51.0 (1.36)
45–64	21.8 (1.40)	32.1 (1.18)	38.1 (1.01)	29.7 (0.76)
65+	8.0 (0.76)	20.7 (1.28)	33.0 (1.50)	19.3 (1.05)
Male	42.4 (0.90)	55.1 (1.19)	49.8 (1.15)	47.9 (0.49)
Race				
White	85.5 (0.86)	84.0 (1.00)	82.7 (1.10)	84.2 (0.80)
Black	10.1 (0.69)	10.2 (0.77)	12.4 (0.95)	10.8 (0.67)
Mexican American	4.4 (0.42)	5.8 (0.54)	5.0 (0.43)	4.9 (0.40)
Marital Status				
Married	69.1 (1.16)	70.6 (1.48)	67.1 (1.03)	68.8 (0.77)
Separated/Divorced	13.8 (0.77)	11.9 (1.09)	12.0 (0.75)	12.7 (0.55)
Single	13.7 (1.16)	11.9 (0.85)	7.0 (0.69)	10.3 (0.66)
Widow	3.7 (0.29)	8.6 (0.66)	14.0 (0.76)	8.2 (0.43)
Education				
<12 years	16.4 (1.03)	25.9 (1.54)	33.5 (1.48)	24.3 (1.11)
High school graduate	32.8 (1.10)	35.4 (1.66)	34.9 (1.32)	35.0 (0.80)
>12 years	50.9 (1.39)	38.7 (2.00)	31.6 (1.65)	41.6 (1.34)
Income				
≤$14,999	13.2 (0.93)	18.5 (1.24)	25.0 (1.26)	18.4 (1.92)
$15,000–24,999	17.9 (1.09)	18.3 (1.23)	19.3 (0.92)	18.5 (0.69)
≥$25,000	64.4 (1.27)	56.8 (2.01)	48.3 (1.69)	57.2 (1.24)
Missing	4.5 (0.46)	6.4 (0.55)	7.5 (0.62)	5.9 (0.35)
**Number of deaths**				
All cause	1,056	1,519	3,451	6,026
Rate per 100,000 person-years	702.2	1699.3	3105.4	1620.7
Cardiovascular Disease	263	486	1,157	1,906
Rate per 100,000 person-years	143.9	478.7	1007.7	468.3

^a^ Proportions and standard errors.

## Results

Consistent with the total population distribution, when compared to participants with an allostatic load score of at least 1, those who have a score of 2 or ≥ 3 were more likely to be older, white, and widowed, but less likely to have a high school diploma, and report an income of less than $15,000 (Table 1). We also observed that mortality rates are directly associated with allostatic load score categories.

High allostatic scores were associated with older age, being black or Mexican American, widowed, less educated and low income (Table 2).

**Table 2:** Prevalence^a^ of allostatic load according to selected socio-demographic and health-related characteristics for adults 25 years of age or older: NHANES III-Linked Mortality File, 2015.

	Allostatic Load Score Categories		
	≤1 (n = 4,699)	2 (n = 3,475)	≥3 (n = 5,499)
Overall	42.7 (1.18)	24.6 (0.73)	32.7 (1.22)
**Socio-Demographic**			
Age			
25–44	58.7 (1.41)	22.7 (0.99)	18.6 (1.21)
45–64	31.4 (1.54)	26.5 (1.08)	42.0 (1.64)
65+	17.7 (1.11)	26.3 (1.19)	55.9 (1.80)
Male	37.8 (1.76)	28.2 (1.01)	34.0 (1.28)
Race			
White	43.3 (1.32)	24.5 (0.86)	32.1 (1.39)
Black	39.7 (1.30)	23.0 (0.85)	37.3 (1.50)
Mexican American	38.3 (1.40)	28.8 (0.92)	32.9 (1.31)
Marital Status			
Married	42.9 (1.33)	25.2 (0.86)	31.9 (1.41)
Separated/Divorced	46.1 (1.78)	23.0 (1.51)	30.9 (1.83)
Single	56.3 (2.58)	21.5 (2.22)	22.2 (1.79)
Widow	19.1 (1.57)	25.5 (1.58)	55.4 (2.16)
Education			
<12 years	28.8 (1.19)	26.1 (1.03)	45.1 (1.47)
High school graduate	41.0 (1.59)	25.5 (1.16)	33.5 (1.71)
>12 years	52.2 (1.56)	22.9 (1.27)	24.9 (1.31)
Income			
≤$14,999	30.7 (1.74)	24.8 (1.31)	44.6 (1.74)
$15,000–24,999	41.5 (2.36)	24.4 (1.43)	34.1 (1.74)
≥$25,000	48.0 (1.33)	24.3 (1.02)	27.6 (1.13)
Missing	32.3 (2.37)	26.5 (1.77)	41.2 (2.53)

^a^ Proportions and standard errors

Participants who died were more likely to be above the at-risk cut points for the allostatic load biomarkers. The mean allostatic load score was significantly higher for those who died than for those who did not die (Table 3).

**Table 3:** Descriptive statistics for biomarkers and allostatic load score for adults 25 years of age or older: NHANES III-Linked Mortality File, 2015.

	Alive (n = 7,647)	Dead^a^ (n = 6,026)	P-Value	Total (n = 13,673)
**Biological indicator** ^b^				
Albumin <3.8 mg/dl	8.2 (0.79)	16.4 (1.61)	<0.01	10.9 (0.90)
C-reactive protein ≥0.3 mg/dl	24.7 (1.27)	41.4 (1.63)	<0.01	30.2 (1.30)
Cholesterol ≥ 240 mg/dl	17.2 (0.74)	29.7 (0.82)	<0.01	21.3 (0.65)
HDL <40 mg/dl	22.5 (0.94)	27.5 (1.21)	0.01	24.2 (0.87)
A1C ≥ 6.4%	2.5 (0.29)	14.5 (0.71)	<0.01	6.4 (0.37)
Waist-to-hip ratio	60.1 (1.23)	86.2 (0.76)	<0.01	68.7 (0.98)
Systolic BP ≥ 140 mmHg	7.3 (0.45)	38.7 (1.26)	<0.01	17.4 (0.80)
Diastolic BP ≥ 90 mmHg	6.6 (0.48)	8.1 (0.49)	0.01	7.1 (0.41)
Pulse ≥ 90 beats/min	9.1 (0.68)	14.0 (0.79)	<0.01	10.6 (0.62)
Allostatic load score	1.57 (0.04)	2.72 (0.05)	<0.01	1.95 (0.04)

^**a**^ Means and standard errors.

^b^ Proportions represent those with values equal or above the ones next to each indicator. For waist-to-hip ratio, the values were >90 for male and >85 for female.

**Fig 1:**
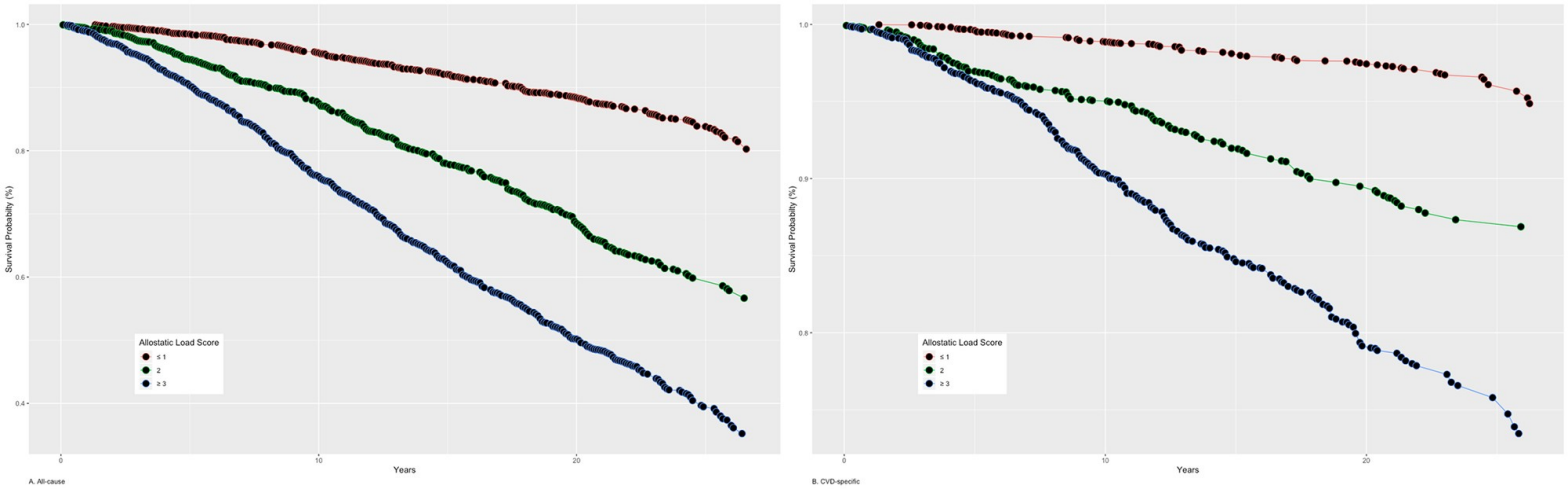
Kaplan-Meier curves for A) all-cause and B) CVD-specific mortality risks according to allostatic load score categories: NHANES III-Linked Mortality File, 2015 (P-values for log-rank test <0.001).

Table 4 presents the unadjusted and adjusted hazard rates for allostatic load scores for all-cause and CVD-specific mortality for NHANES III participants. Compared to participants with an allostatic load score of ≤1, those with an allostatic load score of 2 and ≥3 had a rate of dying of 2.47 (95%CI: 2.20–2.78) and 4.67 (95%CI: 4.22–5.16) times greater, respectively (Fig 1A). The hazard rates were attenuated but remained significant after controlling for age, sex, race/ethnicity, education and income. Regarding CVD-specific mortality, hazard rates increased with high allostatic load scores (Fig 1B). After adjustment for age, sex, race/ethnicity, education and income, the rates of dying for a CVD-specific cause were 1.58 (95%: 1.22, 2.04) and 2.24 (95%CI: 1.82, 2.76) times greater among adults with an allostatic load score of 2 and ≥3, respectively, compared with their counterparts with a score of at least 1.

**Table 4:** Crude and adjusted hazard ratios^a^ of all-cause and CVD-specific mortality risk for allostatic load score categories: NHANES III-Linked Mortality File, 2015.

	Unadjusted	Adjusted
**All-cause**		
Allostatic load score		
≤1	1.00	1.00
2	2.47 (2.20, 2.78)	1.31 (1.17, 1.47)
≥3	4.67 (4.22, 5.16)	1.71 (1.54, 1.90)
**CVD-specific**		
Allostatic load score		
≤1	1.00	1.00
2	3.35 (2.60, 4.31)	1.58 (1.22, 2.04)
≥3	7.12 (5.96, 8.51)	2.24 (1.82, 2.76)

^a^ Adjusted for age, sex, race/ethnicity, education and income.

We found racial/ethnic heterogeneity of the association of allostatic load scores with all-cause mortality (p-interaction = 0.005) and with CVD (p = 0.007). We observed significant associations of allostatic load scores ≥ 3 and all-cause mortality regardless of race/ethnicity (Table 5). However, these associations were stronger among blacks (HR: 1.80; 96%CI: 1.52, 2.14) and whites (HR: 1.72 96%CI: 1.52, 2.14) than Mexican Americans (HR: 1.47; 96%CI: 1.18, 1.82). For CVD, these associations were only observed among whites and blacks with whites having a greater rate of dying associated with allostatic load scores ≥ 3 than blacks. It is worth noting that an allostatic load score of 2 was associated with all-cause and CVD-specific mortality risk among whites only.

**Table 5:** Adjusted hazard ratios^a^ of CVD-specific mortality risk for allostatic load score categories according to racial/ethnic groups: NHANES III-Linked Mortality File, 2015.

	Non-Hispanic White	Non-Hispanic Black	Mexican American
**All-cause**			
Allostatic load score			
≤1	1.00	1.00	1.00
2	1.35 (1.18, 1.54)	1.19 (0.95, 1.49)	0.98 (0.78, 1.25)
≥3	1.72 (1.52, 1.94)	1.80 (1.52, 2.14)	1.47 (1.18, 1.82)
**CVD-specific**			
Allostatic load score			
≤1	1.00	1.00	1.00
2	1.67 (1.26, 2.21)	1.27 (0.88, 1.83)	0.85 (0.47, 1.52)
≥3	2.34 (1.87, 2.92)	1.71 (1.23, 2.40)	1.66 (0.97, 2.84)

^a^Adjusted for age, sex, education and income.

When examining racial/ethnic heterogeneity of the associations between allostatic load scores and mortality outcomes according to age, sex and education, no interaction was observed between race/ethnicity, sex and allostatic load scores (p = 0.20) for all-cause mortality; and race/ethnicity, age groups and allostatic load scores (p = 0.42) and race/ethnicity, education, and allostatic load score (p = 0.62) for CVD-specific mortality. However, we observed interactions between race/ethnicity, age groups and allostatic load score (p = 0.03) and race/ethnicity, education and allostatic load score (p = 0.02) for all cause-mortality; and race/ethnicity, sex and allostatic load score (p = 0.03) for CVD-specific mortality.

Table 6 presents the adjusted hazard ratios and 95% CIs for the association between allostatic load scores and all-cause mortality for each racial/ethnic group stratified by age group and education. For whites ages 25–44 years, the rates of dying for all-cause were 2.31 (95%CI: 1.66, 3.20) and 3.00 (95%CI: 1.89, 4.76) greater than their peers with a score of at least 1. For blacks (HR:2.50; 95%CI: 1.91, 3.29) and Mexican Americans (HR:1.61; 95%CI: 1.22, 2.13) of similar age, the rates of dying were only significant for those with an allostatic load score ≥ 3. For adults ages 45–64 years, higher rates for all-cause mortality were observed for non-Hispanic whites with allostatic load scores 2 and ≥3 (HR:1.40; 95%CI:1.06–1.85 and HR:2.20; 95%CI:1.79–2.71, respectively). However, for blacks and Mexican Americans, the rates of dying were only observed among those with an allostatic load score ≥3. Finally, for those 65 years and older, only whites (HR:1.52; 95%CI: 1.28, 1.81) and blacks (HR:1.25; 95%CI: 1.56, 2.11) had higher rates of all-cause mortality for those with an allostatic load score of ≥3 compared to their peers with a score of ≤1.

**Table 6:** Adjusted hazard ratios of all-cause mortality risk for allostatic load score categories for racial/ethnic groups according to age and education: NHANES III-Linked Mortality File, 2015.

	Non-Hispanic White	Non-Hispanic Black	Mexican American
**Age groups**^a^			
	**Age 25–44**		
Allostatic load score			
≤1	1.00	1.00	1.00
2	2.31 (1.66, 3.20)	1.23 (0.90, 1.67)	0.96 (0.67, 1.39)
≥3	3.00 (1.89, 4.76)	2.50 (1.91, 3.29)	1.61 (1.22, 2.13)
	**Age 45–64**		
Allostatic load score			
≤1	1.00	1.00	1.00
2	1.40 (1.06, 1.85)	1.20 (0.78, 1.85)	1.34 (0.91, 1.97)
≥3	2.20 (1.79, 2.71)	2.22 (1.57, 3.12)	2.32 (1.73, 3.13)
	**Age 65+**		
Allostatic load score			
≤1	1.00	1.00	1.00
2	1.22 (0.99, 1.49)	1.35 (0.92, 1.97)	1.27 (0.77, 2.10)
≥3	1.52 (1.28, 1.81)	1.25 (1.56, 2.11)	1.42 (0.90, 2.25)
**Education**^b^			
	**<12 year**		
Allostatic load score			
≤1	1.00	1.00	1.00
2	1.17 (0.93, 1.47)	1.14 (0.85, 1.55)	0.90 (0.69, 1.17)
≥3	1.58 (1.29, 1.94)	1.67 (1.30, 2.15)	1.36 (1.06, 1.75)
	**High school graduate**		
Allostatic load score			
≤1	1.00	1.00	1.00
2	1.61 (1.26, 2.06)	1.20 (0.83, 1.73)	0.77 (0.39, 1.51)
≥3	1.78 (1.50, 2.11)	2.06 (1.57, 2.70)	1.15 (0.68, 1.96)
	**>12 years**		
Allostatic load score			
≤1	1.00	1.00	1.00
2	1.22 (1.01, 1.47)	1.19 (0.72, 1.98)	1.87 (1.06, 3.32)
≥3	1.72 (1.45, 2.05)	1.63 (1.08, 2.47)	2.83 (1.51, 5.33)

^a^ Adjusted for sex, education and income.

^b^ Adjusted for age, sex and income.

Among adults with less than 12 years of education, the rates of dying were associated with at least a 36% greater rate among adults with an allostatic load score ≥ 3 compared to those with an allostatic load score of ≤1 regardless of race/ethnicity (Table 6). For those who completed a high school degree, the rates of dying were 1.61 and 1.78 times greater for whites with an allostatic load score of 2 and ≥3, respectively, as compared with their counterparts with a score of ≤1. An increased rate of dying was only observed among blacks with an allostatic load score ≥3 (HR:2.06; 95%CI: 1.57, 2.70). Among those with more than 12 years of education, when compared with their counterparts with an allostatic load score of ≤1, non-Hispanic whites and Mexican Americans exhibited higher death rates associated with at least an allostatic load score of 2 whereas for blacks a higher rate was observed for those with a score ≥3.

For sex, white men and women had higher rates of CVD-specific mortality associated with allostatic load scores of at least 2 compared with their counterparts with a score of ≤1 (Table 7). However, hazard rates were stronger among women than among men. For blacks, the association between allostatic load scores and CVD-specific mortality was only significant among women: those with a score of 2 and ≥ 3 had a rate of dying of at least 89% greater than their peers with a score of ≤1.

**Table 7:** Adjusted hazard ratios^a^ of CVD-specific mortality risk for allostatic load score categories for racial/ethnic groups according to sex: NHANES III-Linked Mortality File, 2015.

	Non-Hispanic White	Non-Hispanic Black	Mexican American
	**Men**		
Allostatic load score			
≤1	1.00	1.00	1.00
2	1.81 (1.23, 2.66)	0.96 (0.54, 1.69)	0.78 (0.39, 1.56)
≥3	2.84 (2.11, 3.82)	1.50 (0.92, 2.44)	1.45 (0.82, 2.54)
	**Women**		
Allostatic load score			
≤1	1.00	1.00	1.00
2	1.66 (1.18, 2.33)	1.89 (1.19, 3.01)	0.98 (0.41, 2.33)
≥3	2.05 (1.47, 2.88)	2.20 (1.32, 3.66)	2.28 (0.85, 6.14)

^a^Adjusted for age, education and income

## Discussion

Consistent with previous studies, [12–19] we found that high allostatic load scores are associated with all-cause mortality rates among U.S. adults aged 25 years or older. However, and in contrast to a previous study,[17] we found that high allostatic load scores are associated with CVD-specific mortality and these rates were stronger for CVD-specific than for all-cause mortality. Moreover, we found racial/ethnic heterogeneity of the associations of allostatic load scores with all-cause and CVD-specific mortality risks. However, we observed further racial/ethnic heterogeneity of these associations by age, sex and education. All-cause mortality rates for each racial/ethnic group differed with age and education whereas for CVD-specific mortality rates, this difference was observed for sex only. Allostatic load scores ≥ 3 were associated with an increased rate of dying of any cause among adults younger than 64 years of age regardless of race/ethnicity. However, the rate was stronger for Mexican Americans ages 45–64 years. For adults aged 65 or older, whites and blacks with a score ≥3 had an increased rate of dying compared to their counterparts with a score of at least 1. Among adults with less than a high school education, we found that those with a score of ≥3 had a higher rate of all-cause mortality regardless of race/ethnicity whereas for those with a high school diploma, whites with at least a score of 2 and blacks with a score of ≥3 had higher rates of dying. Allostatic load scores of ≥3 were associated with a greater rate of all-cause mortality among adults with more than 12 years of education regardless of race/ethnicity. However, Mexican Americans had the stronger rate of dying. For CVD-specific mortality, whites with an allostatic load score of at least 2 had higher rates of dying regardless of sex. Rates were stronger for men than for women. For blacks, allostatic load scores of at least 2 were associated with CVD-specific mortality rate among women only.

We found stronger rates of all-cause mortality associated with allostatic load among adults younger than 65 years regardless of race/ethnicity. This finding is consistent with the weathering hypothesis [8] suggesting that allostatic load may have a greater effect on physiological systems at younger ages than later in life. Moreover, the latter may call our attention to the role of chronic stress early in life and its effect on premature death. Interestingly, among adults 65 years or older, this association was only observed among whites and blacks with a score of ≥3.

Low education has been found to be associated with high allostatic load.[23] In addition, education can set an individual socioeconomic trajectory [33] and has a socioeconomic position effect throughout life course.[34] However, no study has examined the role of education on the association between allostatic load scores and mortality risk. Our findings for education across racial/ethnic groups are interesting for several reasons: First, we observed an association between allostatic load scores and all-cause mortality among those with less than a high school education regardless of race/ethnicity. It is worth noting that Mexican Americans exhibited the weakest association. Second, this association was observed among whites and blacks with a high school diploma or GED. Third, while this association was observed among those with an educational attainment greater than 12 years regardless of race/ethnicity, this association was stronger among Mexican Americans. Taken together, these findings underscore the role of socioeconomic position as a fundamental cause or resources that may prevent, reduce or avoid morbidity and premature mortality.[35–37] However, these resources may have a differential effect based on individuals’ race/ethnicity. Our findings show that these resources may not work well in the presence of chronic stress as captured by allostatic load. For instance, the stronger all-cause mortality rates observed among highly educated Mexican Americans suggest that they may not get the same return from education as their white and black counterparts do. The latter may be consistent with the potential explanation of social support associated with the Hispanic paradox and infant mortality.[38] Evidence suggests that social support may be protective against infant mortality risk and may provide a buffer that may protect against environmental stressors.[39, 40] It is possible that highly educated Mexican Americans may lose the benefit of the social connections, and thus, feel isolated. Finally, we found significant associations between allostatic load scores and CVD-specific mortality risk in white men and women as well as in black women. A previous study examining allostatic load and cause-specific mortality among Scottish adults[17] found neither an association between allostatic load with circulatory diseases system nor examined variation by sex or gender.

This study has both limitations and strengths. First, this study used the public-use version of the LMF. When compared to the restricted data, the public data include limited mortality information and small perturbations for a small, selected number of records. However, a comparison of the previous release of the public- and restricted-use linked mortality files found similar results for all-cause and selected cause-specific mortality including CVD-specific mortality.[29] Therefore, our results may not be affected by the use of the publicly available data. Another limitation may be related to the willingness to participate in NHANES III. If individuals who agreed to participate in the NHANES III were different from those who refused to participate, it is possible that our results could be under- or over-estimated. In addition, it is possible that individuals 65 years or older included in the analyses may be different than their counterparts who die, and thus, may introduce survival bias. However, this bias may affect all studies including adults 65 years and older. Among the strengths of this study are the use of a nationally representative sample of non-Hispanic blacks and whites as well as Mexican Americans. Other strengths include the large sample size and number of outcomes. The latter allowed us to control for selected covariates and examine effect measure modifications or statistical interactions. Finally, NHANES included the biomarkers needed to capture allostatic load.

We found that allostatic load score was associated all-cause and CVD-specific mortality among U.S. adults aged 25 years or older, with the strongest associations for CVD-specific mortality. Moreover, we observed racial/ethnic heterogeneity associated with age and education and for all-cause mortality and for sex for CVD-specific mortality. The findings for age and education underscored a stronger effect of allostatic load in young adults and that education may not have the same return across racial/ethnic groups. Mexican Americans ages 45 to 64 and the highly educated carry the greater burden of mortality associated with allostatic load. The stronger associations observed for allostatic load scores and CVD-specific mortality calls attention to the biomarkers used in the score, individually or combined, as red flags for cardiovascular disease morbidity and mortality. Further studies should monitor chronic conditions associated with the biomarkers used to define allostatic load to identify high-risk groups to help monitor social inequities in mortality risk, especially premature mortality.
